# Effectiveness of Valsartan’s Single-Pill Combination Therapies on Blood Pressure Control in Hypertensive Patients: Malaysian Single-Centre Real-World Experience

**DOI:** 10.21315/mjms2023.30.5.10

**Published:** 2023-10-30

**Authors:** Ahmad K. M. Yusof, Norhazreen Mohamad Halil, Norfazlina Jaffar, Intan Safarinaz Sabian, Zhi Ling Looi

**Affiliations:** 1 Cardiology Department, Institut Jantung Negara, Kuala Lumpur, Malaysia; 2 Data Management and Biostatistical Support, Clinical Research Department, Institut Jantung Negara, Kuala Lumpur, Malaysia; 3 Medical Affairs, Novartis Malaysia, Selangor, Malaysia

**Keywords:** single-pill combination, blood pressure, hypertension, valsartan-based, drug combination

## Abstract

**Background:**

Uncontrolled hypertension can cause cardiovascular disease and is an important public health issue. Single-pill combination (SPC) therapies possess combined blood pressure (BP)-lowering effect and may improve compliance to treatment. This study assessed the effectiveness of valsartan (Val)-based SPC therapies in achieving BP control in hypertensive patients.

**Methods:**

This was a retrospective study. Data were extracted from the hybrid medical records of patients from the Institut Jantung Negara (IJN), Malaysia. Adults with established diagnosis of hypertension and on prescription of Val-based SPC therapies as part of routine medical care from 1 January 2013 to 31 December 2018, with ≥ 1 year of follow-up were included. Primary endpoint was proportion of patients achieving therapeutic BP control (BP < 140/90 mmHg). Secondary outcomes included change from baseline (CFB) in systolic BP (SBP) and diastolic BP (DBP), and subgroup analysis was based on baseline SBP categories and presence of diabetes.

**Results:**

Study included 409 hypertensive patients. The mean (standard deviation [SD]) age of the population was 65.1 (10.6) years old, with male predominance (61.6%). Proportion of patients achieving target BP between baseline and follow-up were 57.0% (*P* < 0.001). Mean CFB in SBP and DBP were recorded as 19.52 mmHg and 7.47 mmHg, respectively. Over half of the patients achieved the target BP in all subgroups categorised by SBP at baseline, except the subgroup of SBP 160 mmHg–179 mmHg. SPC therapies were continued in 97.3% of patients at 1-year follow-up.

**Conclusion:**

Patients using Val-based SPC therapies had significant reduction in BP with good tolerability, with 57% of patients achieving target BP over a prolonged 1-year follow-up period. Uptake of SPC therapy is warranted to improve patient care and outcomes in hypertension.

## Introduction

Globally, an estimated 1.13 billion people have hypertension, two-thirds of whom reside in low- and middle-income countries (1). Globally, the proportion of hypertensive patients achieving blood pressure (BP) control in 2010 was 13.8%, which was further reduced to 7.7% in low- and middle-income countries (2). Less than a quarter of hypertensive patients had their hypertension under control in the United States (US), as reported by the Centres for Disease Control and Prevention for data collected from 2015 to 2018 (3). Uncontrolled hypertension is known to contribute to the development of cardiovascular disease (CVD), leading to stroke, heart failure (HF), coronary artery disease and kidney disease (4). More aggressive therapeutic targets for BP control (< 130/80 mmHg) in hypertensive patients from the American College of Cardiology/American Heart Association guidelines acknowledge the increased risk associated with uncontrolled BP (4, 5). The 2018 European Society of Cardiology/European Society of Hypertension (ESC/ESH) guidelines for the management of hypertension identified a BP goal of < 140/90 mmHg in all patients and recommended that treated BP values should be targeted to 130/80 mmHg or lower (6). From 1990 to 2015, the estimated rate of annual deaths for those having systolic blood pressure (SBP) ≥ 140 mmHg increased from 97.9 to 106.3 per 100,000 persons (7). Evidently, hypertension has a considerable impact on morbidity and mortality in adults (8, 9). In addition to the clinical burden, the disease also contributes significantly to the economic burden on patients. In low- and middle-income countries, it is the third leading cause of attributable burden of disease (5.6% of disability-adjusted life years) (10). In Malaysia, the direct cost to the Ministry of Health for antihypertensive medication in 2016 was reported as RM608.8 million—an increase from RM570.3 million in 2014 (11).

In one of the recently conducted Malaysian surveys, The National Health and Morbidity Survey (NHMS) 2019, the overall prevalence of hypertension in adults aged ≥ 18 years old was 30.0%. In the population surveyed, with increase in age, the prevalence of hypertension increased and was as high as 81.7% in patients aged ≥ 75 years old (12). Of all hypertensive patients, only half were aware of having the disease and 90% were taking medications for the same (13). According to the Malaysian Burden of Disease and Injury Study (2009–2014), approximately 34.8% of deaths in Malaysia were attributed to cardiovascular and circulatory diseases (12). Thus, it can be inferred that hypertension is an important medical and public health issue in Malaysia.

Hypertension is managed using both pharmacological and non-pharmacological options. Pharmacological treatment is aimed at reducing BP which assists in reducing the cardiovascular risk in hypertensive patients. Initiation of drug treatment is recommended in patients with stage I hypertension with medium risk per the 2018 Clinical Practice Guidelines on Management of Hypertension in Malaysia (11).

Guidelines mostly recommend monotherapy in patients with stage I hypertension (5, 11, 14), followed by either an increase in the dose of the drug of choice, substitution of the class of drug used or using a single-pill combination (SPC) therapy (11). In the Malaysian population, less than half of the patients on hypertensive medication had their BP under control (13). A cross-sectional study conducted in Malaysia reported that only 32.4% of patients with hypertension were on medication and only 26.8% of patients achieved BP control (15). Low treatment adherence has also been reported in hypertensive patients, with over 50% treatment discontinuation occurring within 1 year or more (16, 17).

In recent times, device-based therapies to reduce BP have also been investigated, of which catheter-based renal denervation (RDN) therapies have been extensively studied (18). RDN acts selectively by interrupting the afferent and efferent nerves of the sympathetic system which leads to a reduction in BP. The various methods for RDN include radiofrequency ablation, ultrasound ablation and chemical ablation (19). The Malaysian guidelines recommend that such device-based therapies are not used in routine medical care and further investigations are required to shed light on predictors of a good response to such treatment (11).

Prescribing a SPC therapy provides the benefit of having an additive BP-lowering effect along with reduced adverse effects, as individual drugs in the SPC therapy are within their tolerable dose range (17). BP control is achieved quickly and this regimen may help reduce the pill burden in these patients as medications are indicated for chronic use (11). The usual combinations of two or three antihypertensive medications include renin-angiotensin-aldosterone system (RAAS) blocker with calcium channel blocker (CCB) and RAAS blocker plus CCB coupled with diuretic, respectively (6, 11, 18, 19).

The objective of this study was to provide real-world evidence on the effectiveness of amlodipine/valsartan (Aml/Val) and amlodipine/ valsartan/hydrochlorothiazide (Aml/Val/HCTZ) SPC therapies for achieving BP control (Valsartan Single Pill-Combination Therapy: Real-World Experience [VaREAL] study) in patients with hypertension from 2013 to 2017 at the Institut Jantung Negara (IJN) (also known as the National Heart Institute) in Malaysia.

## Methods

Data for this retrospective, observational cohort study were collected from the hybrid medical record, that is, physical and electronic medical records of the patients at IJN, Malaysia, which specialises in cardiovascular and thoracic medical and surgical treatments of adult and paediatric patients. IJN is, also, recognised as one of the leading centres in the region. Malaysia is a country which hosts people from different races and religions, with the major races being Malays, Chinese and Indians (20). The IJN is a tertiary referral centre for patients from across Malaysia; thus, this centre addresses the population characteristics of the inter-racial presence in the nation and is a representative of the population.

The data collection period for identification of patients was from 1 January 2013 to 31 December 2017. The 1-year follow-up period lasted until 31 December 2018. The overall study period lasted from 1 January 2013 to 31 December 2018. Patients with hypertension who were prescribed a SPC therapy of Aml/Val or Aml/Val/HCTZ (irrespective of the strength) at the centre were eligible for inclusion. Data collection variables included demographics (age, gender and body mass index), medical history and risk factors (CVD, ischaemic stroke, cerebral haemorrhage, transient ischaemic attack, angina pectoris, congestive HF, diabetes mellitus [DM], smoking, plasma cholesterol > 5.72 mmol/L, family history of DM and evidence of target organ injury) and investigations of SBP and diastolic blood pressure (DBP). The information was stored in a data collection form.

The detailed criteria for inclusion of participants into the study were as follow:

- Adults aged ≥ 18 years old with an established diagnosis of hypertension
- Patients who were treated with Aml/Val or Aml/Val/HCTZ SPC therapies by their treating physicians as part of routine clinical practice
- Patients who completed 12 months on the treatment

Data from at least two visits were recorded (baseline: before commencement of SPC therapy; final: 31 December 2018). The exclusion criteria were as follow:

- Patients who received any other antihypertensive combination therapy prior to the start of the Val-based combination
- Patients who received steroids or traditional Chinese medicines for > 1 week prior to the start of the Val-based combination
- Patients diagnosed with diseases requiring steroid administration prior to the start of Val and its combination therapies
- Patients on other combination therapies, including antihypertensive medication used in the study
- Patients with any contraindication to treatments per the local prescribing information

### Study Outcomes

The primary endpoint was the proportion of patients on Aml/Val and Aml/Val/HCTZ SPC therapy who achieved the therapeutic BP control target defined as SBP < 140 mmHg and DBP < 90 mmHg at 12 months of treatment with the SPC therapy. All thresholds for therapeutic BP control were in accordance with the 2018 Clinical Practice Guidelines on Management of Hypertension in Malaysia (11).

The secondary outcome included change from baseline (CFB) in mean SBP and mean DBP. A subgroup analysis was conducted for the outcome of CFB based on baseline SBP: i) 140 mmHg–159 mmHg, ii) 160 mmHg– 179 mmHg and iii) ≥ 180 mmHg. An additional subgroup analysis was conducted in patients with comorbid diabetes to determine the outcome of proportion of patients achieving therapeutic BP control, defined as SBP < 140 mmHg and DBP < 80 mmHg.

Safety and tolerability were assessed based on the occurrence of adverse events and serious adverse events as reported in the medical records.

### Statistical Analysis

Continuous variables for normally distributed data were presented as mean with standard deviation (SD) and as median with interquartile range for data not normally distributed for baseline characteristics. Categorical variables were presented as frequencies with percentages. For the primary endpoint analysis, categorical variables were analysed using the Pearson’s chi-square test. A *P*-value < 0.05 was considered statistically significant. Patients with the proportion of comorbidities occurring between baseline and follow-up were assessed using the McNemar’s test. All analyses were conducted using the IBM SPSS Statistics for Windows, version 27.0 (IBM Corp., Armonk, NY, USA). For secondary endpoints, descriptive statistics were used to summarise the study variables.

## Results

Based on the inclusion and exclusion criteria, a total of 409 patients were included in the final analysis. Table 1 describes the demographic characteristics of the study population. The mean ± SD age of the population was 65.1 ± 10.6 years old, with a male predominance (61.6% males; 38.4% females), and 55.4% of patients had hypertension as their primary diagnosis. The mean duration from first hypertension drug prescribed in IJN to VaREAL study year was 7.4 (± 4.4) years. The proportion of patients utilising the various investigated SPC therapies were: i) Aml/Val (10/160): 52.1%; ii) Aml/Val (5/80): 40.3% and iii) Aml/Val/HCTZ (10/160/12.5): 7.6%.

Table 2 shows the clinical characteristics of the study population. A high incidence of hyperlipidaemia, CVD and DM was observed in the population. The occurrence of angina pectoris over the follow-up of 1 year was significantly greater than that at baseline (3.4% versus 0.5%), whereas for the other comorbidities, no statistically significant difference was observed (Table 2).

### Attaining Blood Pressure Control

Compared with baseline, BP control (target BP goal of < 140/90 mmHg) achieved in patients after 1 year of treatment corresponded to 4.2 times of change. The data showed a statistically significant difference in patients achieving control of BP over the 1 year of follow-up where the proportion rose from 13.7% at baseline to 57% at follow-up (*P* < 0.001).

### Change in Sistolic Blood Pressure and Diastolic Blood Pressure

**Figure 1:**
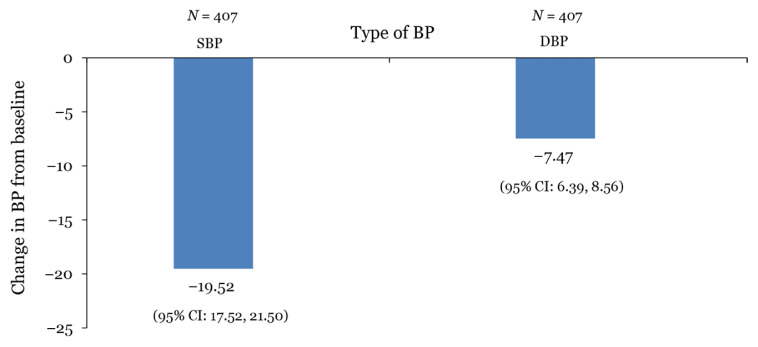
Change in BP from baseline until 1-year of follow-up Notes: BP = blood pressure; CI = confidence interval; DBP = diastolic blood pressure; SBP = systolic blood pressure

By analysis of the 407 patients who had two observations of BP, mean SBP at baseline decreased from 155.88 mmHg (95% confidence interval [CI]: 154.16, 157.59) to 136.11 mmHg at 1 year of follow-up (95% CI: 134.47, 137.75). Similarly, DBP at baseline decreased from 82.57 mmHg (95% CI: 81.45, 83.69) to 74.97 mmHg at 1 year (95% CI: 74.01, 75.94). Both SBP and DBP were significantly reduced over the 1-year follow-up period (*P* < 0.001, using paired samples test). Figure 1 shows the mean difference in SBP and DBP from baseline to 1 year of follow-up (*n* = 407).

The change in BP according to deciles are presented in Table 3. For both SBP and DBP, > 20.0% of patients showed a reduction in the 11 mmHg–20 mmHg range, 26.2% and 23.0%, respectively. Few patients showed an increase in SBP and DBP over the follow-up period (SBP: 14.2%; DBP: 21.0%). The majority of patients had a reduction in their BP, showing the effectiveness of the treatments in reducing both SBP (84.1%) and DBP (74.8%).

### Subgroup Analysis

**Figure 2:**
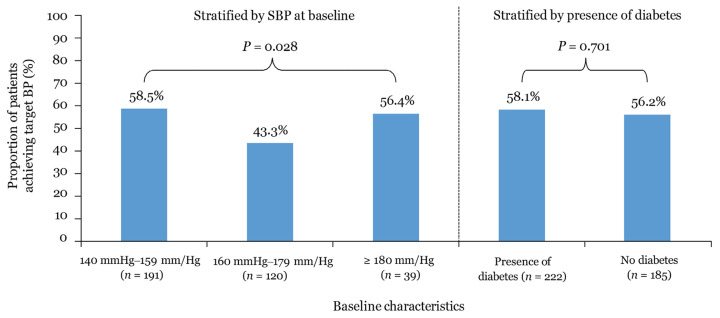
Proportion of patients achieving target BP over the 1-year follow-up Notes: BP = blood pressure; SBP = systolic blood pressure

Two subgroups were evaluated after stratifying the patients according to baseline SBP and presence of DM at baseline. The BP control outcome was defined as achieving a target BP of < 140/90 mmHg in the SBP subgroups and < 140/80 mmHg in patients with diabetic comorbidity at the 1-year follow-up. The results were statistically significant among the subgroups stratified by SBP at baseline (*P* = 0.028), whereas the presence or absence of DM at baseline did not show any statistically significant differences (*P* = 0.701) (Figure 2). In all SBP subgroups, except for the baseline SBP 160 mmHg–179 mmHg subgroup, over half of the population achieved target BP control after 1 year of follow-up.

### Safety and Tolerability

The SPC therapies were fairly tolerated, as 97.3% of the patients continued the medications and 98.0% had no major adverse cardiovascular event (MACE). Hypotension was reported in 0.2% of patients as the reason for treatment discontinuation, whereas 0.5% of patients did not state any reason and 2.0% of patients switched to another drug. Five deaths were reported over the 1-year follow-up. The proportion of patients affected as part of MACE was 0.2% and 1.0% by stroke and myocardial infarction, respectively, whereas 0.5% of patients had cardiac death and 0.2% had noncardiac death.

## Discussion

The patient profile observed in our study was somewhat similar to that observed in two other cross-sectional studies conducted in Malaysia (21, 22). The mean age of the population in these studies was ≥ 61 years old, whereas our study population had a mean age of 65.1 years old. As opposed to our study, a predominance of females (60.4%) was reported in the study by Teh et al. (21), which used data from only public primary care clinics in Malaysia.

This real-world study found that the use of Val-based SPC therapies led to a significant attainment of therapeutic BP control in as many as 57% of the population over the 1-year followup. The results were comparatively better than those from the NHMS Survey 2019 (45%) (13) and from a cross-sectional study of patients diagnosed with hypertension (*n* = 13,784) in public primary care clinics in Malaysia, where an adequate BP control was achieved in 42.8% of the population (21). In contrast, BP control was attained in > 60% of the patients across four observational studies—two from China (23, 24) that assessed Aml/Val SPC therapy and two multinational studies that evaluated Aml/Val SPC therapy (25) and Val/HCTZ SPC therapy (26). However, this variability could be attributed to the varied follow-up time periods and patient populations across these studies (Table 4) compared to our study. The importance of SPC therapies in achieving target BP control has been highlighted in few studies (Table 4). A recent systematic rapid assessment review of evidence published from 1 January 2013 to 11 January 2019 analysed the effect of using SPC therapy on adherence, BP control and clinical outcomes in arterial hypertension. Nine out of the ten studies reporting BP goal attainment versus baseline showed that ≥ 50% of patients achieved the goal within 6 months of the follow-up period (27). Similarly, in the clinical EXperienCe of amlodIpine and valsarTan in hypErtension (EXCITE) study and its interim results (28, 29) during the 26-week follow-up period, > 50% of patients attained their BP goals. Our study observed that the presence or absence of DM as a comorbidity at baseline did not cause any significant difference in change in BP control. These findings were in line with the China Survey of hyperTensive pAtienTs blood pressUre control rate in clinic Service (STATUS) II study, which inferred that the BP-lowering efficacy of Aml/Val SPC therapy is independent of age and comorbidities (30). Over the follow-up period of 1 year, the percentage CFB reductions in SBP and DBP observed in our study were 12.5% and 9.1%, respectively. In a study by Zhang et al. (23), at 8 weeks, the Aml/Val SPC therapy significantly (*P* < 0.0001) reduced SBP and DBP in 85.7% and 64.3% of patients, respectively. In another multinational study by Chazova et al. (31), hypertensive patients taking free-dose Aml/Val combinations showed significant percent CFB reductions in SBP and DBP, 20.4% and 17.6%, respectively, after 3 months of follow-up. Similar findings with significant reductions from baseline were observed in the literature studies with varied VAL SPC therapies and follow-up durations (Table 4) (24–26, 28–30, 32–34).

The use of VAL-based SPC therapies in our study also showed good tolerability, with only 2.7% discontinuations observed. In our study, hypotension was reported as a reason of discontinuation in only one patient. Only 2% of patients had an occurrence of MACE. Of note, this study was not designed as a safety study, and the outcomes were collected as reported in the medical records. In a systematic review by Tsioufis et al. (27), hypotension and oedema were identified as common adverse events. The proportion of patients reporting serious adverse events was less than 1%, indicating tolerable profile of various SPC therapies used for treating hypertension (27).

In a systematic review assessing the impact of SPC therapies on clinical outcomes, 9 out of 14 observational studies comparing the adherence or persistence in hypertensive patients using SPC therapies as opposed to free-dose combinations showed significantly better results in favour of SPC therapies (27). This underscores the importance of SPC therapies in achieving the target BP owing to patient adherence and ensuring the use of tolerable doses of individual components in the SPC therapy.

The use of SPC therapies to achieve favourable outcomes for patients by improving BP control, reducing pill burden and increasing patient compliance has been well understood (27, 35). The 2018 ESC/ESH guidelines on the treatment of arterial hypertension recommend initiation of antihypertensive treatment in all patients by using two-drug SPC therapy, with the exception of frail older patients, low-risk and grade I hypertension patients (especially when SBP < 150 mmHg) (6). In the American guidelines, however, treatment may be initiated with two first-line antihypertensive agents as separate drugs or fixed-dose combinations in stage II hypertension patients with an average BP of 20/10 mmHg more than the BP target (6, 35). In Malaysia, the guidelines recommend the use of combination therapy as a free-drug combination or SPC therapy (11). The guidelines acknowledge that evidence from the literature supports the overall benefit of using SPC therapies as opposed to free-dose combinations. Furthermore, the Malaysian guidelines emphasise that the use of SPC therapies not only improves adherence but also lowers the overall healthcare cost (11).

Apart from the use of pharmacological treatments, there is growing evidence of devicebased treatments, such as RDN, to control elevated BP. However, there are some challenges associated with these interventions. Primarily, guidelines do not recommend the use of RDN in routine clinical practice but only in cases of uncontrolled or resistant hypertension (6, 11). These procedural interventions require skill and expertise are invasive and may provide variable responses. Hence, it is important to optimise these processes before encouraging its adoption (18, 36). Data on the long-term effect of the RDN on renal innervation and the maintenance of antihypertensive efficacy are still scarce and warrant further research. Owing to these constraints, the acceptability and reliability of RDN may be questionable.

The strength of our study includes the display of the significant beneficial effect of using SPC therapies in the hypertensive population to achieve the target BP for a long duration (1 year). However, this study has certain limitations. Considering the retrospective nature of data collection, it may be prone to misclassification, recall bias or data collection errors. Additionally, because the data were collected from a real-world registry, the 1-year follow-up data were not structured and planned as observed in clinical trials. Although this was a single-centre study, we believe that it presents realworld clinical practice from a large tertiary referral centre; thus, these results may be generalisable to the Malaysian population.

## Conclusion

This study highlights the effectiveness of Val-based SPC therapies in treating hypertension in a real-world clinical practice over a follow-up of 1 year. The proportion of patients in this study who achieved therapeutic BP control (57%) was much higher than that reported in the literature. Thus, SPC therapies should be considered as the preferred option for treating hypertensive patients.

## Figures and Tables

**Table 1:** Characteristics of the hypertensive patients included in the study (*n* = 409)

Characteristic	Total baseline sample (*n* = 409)	Follow-up data at 1 year (*n* = 409)	*P*-value
Age, mean ± SD years old	65.13 ± 10.63	–	–
Males, *n* (%)	252 (61.6)	–	–
Body mass index^a^, mean ± SD	29.58 ± 4.22	29.18 ± 4.24	0.184
SBP, mmHg			
Mean ± SD	155.88 ± 17.65	136.11 ± 16.86	< 0.001
Median (IQR)	155.00 (145.00, 165.00)	136.00 (125.00, 147.00)	–
DBP, mmHg			
Mean ± SD	82.57 ± 11.51	74.97 ± 9.91	< 0.001
Median (IQR)	82.00 (75.00, 90.00)	75.00 (69.00, 81.00)	–
Race/ethnicity, *n* (%)			
Malay	212 (52.0)	–	–
Chinese	74 (18.0)	–	–
Indian	101 (25.0)	–	–
Other Malaysian	18 (4.0)	–	–
Foreigner	4 (1.0)	–	–
Primary diagnosis, *n* (%)			
Arrythmia	15 (3.7)	–	–
DM	15 (3.7)	–	–
Dyslipidaemia	8 (2)	–	–
Hypertension	223 (54.5)	–	–
IHD	147 (35.9)	–	–
PAD	1 (0.2)	–	–
Duration of disease related to hypertension, years^b^	7.4 ± 4.4	–	–

Notes:

a Data presented for only 31 patients;

b Duration from the first HTN drug prescribed in IJN to VaREAL study year;

DBP = diastolic blood pressure; DM = diabetes mellitus; HTN = hypertension; IHD = ischaemic heart disease; IJN = Institut Jantung Negara; IQR = interquartile range; PAD = peripheral arterial disease; SBP = systolic blood pressure; SD = standard deviation

**Table 2:** Clinical characteristics of hypertensive patients included in the study (*n* = 409)

Clinical characteristic	Total baseline sample (*n* = 409)	Follow-up data at 1 year (*n* = 409)	*P*-value
Comorbidities, *n* (%)			
Smoking	5 (1.2)	4 (1.0)	1.000
CVD	231 (56.5)	235 (57.5)	0.508
Stroke, ischaemic	6 (1.5)	9 (2.2)	0.250
Cerebral haemorrhage	0 (0.0)	1 (0.2)	1.000
TIA	0 (0.0)	1 (0.2)	1.000
Angina pectoris	2 (0.5)	14 (3.4)	0.002
Diabetes mellitus	223 (54.5)	226 (55.3)	0.508
Hyperlipidaemia	181 (44.3)	187 (45.7)	0.180
Family history of diabetes	5 (1.2)	6 (1.5)	1.000
Evidence of target organ injury	3 (0.7)	6 (1.5)	0.250

Notes: CVD = cardiovascular disease; TIA = transient ischaemic attack

**Table 3:** Proportion of patients (reported in %) showing change in BP from baseline to follow-up according to deciles

Δ, mmHg	< 10	11–20	21–30	31–40	41–50	51–60	> 60	Increasing	No change	No reading
SBP	16.6	26.2	17.1	10.5	4.9	5.4	3.4	14.2	1.2	0.5
DBP	41.1	23.0	8.1	1.5	1.0	0.2	–	21.0	3.7	0.5

Notes: BP = blood pressure; DBP = diastolic blood pressure; SBP = systolic blood pressure

**Table 4:** Comparative assessment from literature studies

Author and year	Country	Characteristics of subjects	Sample size	Research design	Findings
Chazova et al., 2011 (31)	Multinational (Asia, Egypt and Russia)	Adult patients with arterial HTN	2,729	Prospective; open label	At 12 weeks, the free-dose combination of Aml/Val showed effective mean SBP reduction of −55 from the baseline.
Lai et al., 2011 (26)	Multinational (Asia)	Patients with stage 1/2 essential HTN with at least one dose of Val/HCTZ SPC therapy	7,567	Observational	At week 24, Val/HCTZ SPC therapy significantly reduced SBP and DBP from baseline −25.4 ± 15.2 mmHg and −14.9 ± 13.5 mmHg; *P* < 0.001, respectively. Response and BP control rates increased continuously from baseline in 94.6% and 73.2% of patients, respectively.
Karpov et al., 2012 (25)	Multinational (Asia, Middle East and Russia)	Adults with arterial HTN	8,336	Prospective; open label	At week 12, Aml/Val SPC therapy significantly reduced mean BP from baseline 165.0/99.3 mmHg to 128.7/80.4 mmHg (−36.3/−18.9 mmHg), P < 0.0001. BP control was achieved in 77.7% of patients.
Hu et al., 2014 (China STATUS II) (30)	China	Patients aged ≥ 18 years old with essential HTN	11,422	Prospective; open label	At 8 weeks, Val/Aml SPC therapy combination showed a significant reduction from baseline in msSBP of −27.1 mmHg (159.6 versus 132.5 mmHg; P < 0.0001) and in msDBP of −15.2 mmHg (95.6 versus 80.4 mmHg). The BP-lowering efficacy of Val/Aml SPC therapy was independent of age and comorbidities.
Khan et al., 2014 (29)	Pakistan	Patients aged ≥ 18 years old with HTN	500	Prospective; open label	At week 26, in the Aml/Val cohort, the mean BP decreased from baseline 153.4/91.1 mmHg to 128.9/78.4 mmHg (−24.5/−12.7 mmHg; *P* < 0.0001). In the Aml/Val/HCTZ cohort, the mean BP decreased from 171.6/99.3 mmHg to 127.7/77.4 mmHg (−43.9/−21.9 mmHg; P < 0.0001). The BP goal was attained by 57% of patients in the Aml/Val cohort and 55.2% of patients in the Aml/Val/HCTZ cohort.
Sison et al., 2014 (EXCITE study) (28)	Multinational (Asia and Middle East)	Adult patients with HTN	9,794	Prospective; open label	i)At week 26 ± 8 weeks, both Aml/Val and Aml/Val/HCTZ SPC therapies provided statistically significant reductions in msSBP (95% CI) from baseline, −31.0 (−31.42, −30.67) mmHg and −36.6 (−37.61, −35.50), respectively.ii)BP goals were achieved in 52.8% of patients receiving Aml/Val and 54.5% of patients receiving Aml/Val/HCTZ.
Ge et al., 2015 (24)	China	Adults aged ≥ 18 years old with essential HTN with excess body weight and uncontrolled by monotherapy	11,312	Prospective; open label	At 8 weeks, Val/Aml SPC therapy reported significant (*P* < 0.001) msSBP/msDBP reductions from baseline across all BMIs (normal, overweight and obese patients) and WC subgroups. BP control with Val/Aml SPC therapy was achieved in 64.5% of men and 64.4% of women.
Setiawati et al., 2015 (33)	Egypt	Patients ≥ 18 years old with essential hypertension	500	Prospective; open label	At week 26, Aml/Val SPC therapy showed effective msSBP and msDBP reductions (95% CI) from baseline −33.7 (−35.2, −32.1) mmHg and −14.8 (−15.7, −13.8) mmHg, respectively.
Assaad-Khalil and Nashaat, 2016 (EXCITE study) (32)	Egypt	Patients aged ≥ 18 years old with HTN who received the Aml/Val SPC therapy either as single therapy or add-on therapy	2,566	Prospective	At week 26, Aml/Val SPC therapy provided clinically significant (*P* < 0.0001) BP reductions in msSBP/msDBP from baseline by −34.5/−19.4 mmHg, respectively.
Wang and Chen, 2016 (34)	China	Adults aged ≥ 18 years old with essential HTN and uncontrolled by monotherapy	11,312	Prospective; open label	At 8 weeks, significantly higher proportion of women compared to men achieved the target BP (77.78% versus 76.22%, *P* < 0.05, respectively) with Val/Aml SPC therapy.
Zhang et al., 2017 (China STATUS II sub-analysis) (23)	China	Patients with HTN and different types of strokes	565	Prospective	At 8 weeks, Val/Aml SPC therapy significantly reduced msSBP/msDBP from baseline (≥ 20/10) in 85.7% and 64.3% of patients (*P* < 0.0001). BP control was achieved in 92.9% of patients.
Yusof et al., 2023 (present study)	Malaysia	Adults with HTN	409	Retrospective	At 52 weeks, Aml/Val or Aml/Val/HCTZ SPC therapies significantly (*P* < 0.001) reduced SBP/DBP from baseline by −19.52/−7.47 mmHg. BP control was achieved in 57% of patients. The presence or absence of DM as a comorbidity at baseline did not bring any significant difference in BP control.

Notes: Aml = amlodipine; BMI = body mass index; BP = blood pressure; CI = confidence interval; DM = diabetes mellitus; HCTZ = hydrochlorothiazide; HTN = hypertension; msDBP = mean sitting diastolic blood pressure; msSBP = mean sitting systolic blood pressure; SPC = single-pill combination; Val = valsartan; WC = waist circumference
